# Predictors of Intensive Treatment in Patients With Obsessive-Compulsive Disorder

**DOI:** 10.3389/fpsyt.2021.659401

**Published:** 2021-04-12

**Authors:** Johanna A. M. du Mortier, Karin C. P. Remmerswaal, Neeltje M. Batelaan, Henny A. D. Visser, Jos W. R. Twisk, Patricia van Oppen, Anton J. L. M. van Balkom

**Affiliations:** ^1^GGz Centraal, Innova, Amersfoort, Netherlands; ^2^Amsterdam UMC, Vrije Universiteit, Department of Psychiatry, Amsterdam Public Health Institute and GGZ inGeest Specialized Mental Health Care, Amsterdam, Netherlands; ^3^Amsterdam UMC, Vrije Universiteit, Epidemiology and Biostatistics, Amsterdam, Netherlands

**Keywords:** obsessive-compulsive disorder, OCD, intensive treatment, longitudinal, quality of life, psychotropic medication, comorbid depression

## Abstract

**Background:** Few studies have investigated which patients with obsessive-compulsive disorder (OCD) do not recover through regular cognitive behavior therapy or pharmacotherapy and subsequently end up in intensive treatment like day treatment or inpatient treatment. Knowing the predictors of intensive treatment in these patients is significant because it could prevent intensive treatment. This study has identified predictors of intensive treatment in patients with OCD.

**Methods:** Using 6-year longitudinal data of the Netherlands Obsessive Compulsive Disorder Association (NOCDA), potential predictors of intensive treatment were assessed in patients with OCD (*n* = 419). Intensive treatment was assessed using the Treatment Inventory Costs in Patients with Psychiatric Disorders (TIC-P). Examined potential predictors were: sociodemographics, and clinical and psychosocial characteristics. Logistic Generalized Estimating Equations was used to estimate to what extent the various characteristics (at baseline, 2- and 4-year assessment) predicted intensive treatment in the following 2 years, averaged over the three assessment periods.

**Results:** Being single, more severe comorbid depression, use of psychotropic medication, and a low quality of life predicted intensive treatment in the following 2 years.

**Conclusions:** Therapists should be aware that patients with OCD who are single, who have more severe comorbid depression, who use psychotropic medication, and who have a low quality of life or a drop in quality of life are at risk for intensive treatment. Intensive treatment might be prevented by focusing regular treatment not only on OCD symptoms but also on comorbid depression and on quality of life. Intensive treatment might be improved by providing extra support in treatment or by adjusting treatment to impairments due to comorbid depressive symptoms or a low quality of life.

## Introduction

Obsessive-compulsive disorder (OCD) is an impairing disorder, often with a chronic course (1). There are evidence-based treatments for OCD, namely cognitive behavior therapy (CBT) and psychotropic medication, that can be offered in more or less intensive formats (2). Multidisciplinary guidelines recommend determining the designated intensity of treatment according to the principles of so-called “stepped care” (3–5). In line with stepped care, the least intensive treatment possible is delivered to patients first, taking into account the nature and course of their symptoms. In the case of non-response, treatment may be “stepped up” to a more intensive level in an effort to meet the treatment goals (6). In the National Institute for Health and Care Excellence (NICE) guideline, the first step in the treatment of OCD consists of awareness, recognition and assessment (3). Next step strategies comprise of CBT, antidepressant medication, or a combination of these. In the case of non-response, treatment is stepped up to treatment by a multidisciplinary team with expertise in the management of OCD. Intensive treatment such as day treatment or inpatient treatment may be considered in this latter step for the most severe, impaired, and treatment-resistant patients. In the Netherlands, intensive treatment usually consists of multimodal treatment, with CBT being the main therapy, offered in a group with other patients with anxiety disorders and OCD. It can be offered in a day-care setting or in an inpatient setting. Intensive treatment usually takes several (parts of) days a week up to 5 days a week for a few months to 1 year. Admission may also be necessary when patients are in crisis.

Up till now, no longitudinal studies into the predicting factors of intensive treatment in OCD have been published. However, cross-sectional research exists, describing the characteristics of patients with OCD in intensive residential treatment. These patients were treatment-resistant to antidepressants and/or CBT, suffered from severe OCD symptoms and psychiatric comorbidity (7–9), had an early age of onset of OCD and a long duration of the disorder (10, 11), often did not have a partner or a job (10–12) and had a low quality of life, with scores of one to two standard deviations below the general population (13–17).

In populations with other mental disorders, more is known about predictors of hospitalization. A systematic review of 58 papers on predictors of readmission in patients with several mental disorders indicates that previous hospitalization, younger age, being unmarried, having lower financial means, not being satisfied with the index treatment, having more hospital days on the index admission, and a negative attitude toward medication were predicting factors for psychiatric readmission (18). In addition, being male, having psychotic symptoms, a longer duration of untreated psychosis, less social satisfaction, disturbed family dynamics, residing in an urban area, and illegal drugs misuse were found predictive of hospitalization in recent prospective cohort studies involving several mental disorders (18–22). A population study combined several survey and register databases of 2,638 individuals born in 1953, including interviews with their mothers. From this study it appeared that poor family relations in adolescence were associated with an increased risk of inpatient psychiatric treatment in the years 1969 until 2008 (20).

Knowledge of characteristics that predict future intensive treatment might help to optimize first steps of treatment for patients with OCD to prevent the need for intensive treatment. This is significant because intensive treatment may contribute to stigmatization and the disruption of the lives of patients by hindering work, education, care for children, hobbies or social contacts (23, 24). In addition, intensive treatment is expensive, which burdens society with costs. Nevertheless, intensive treatment is still the best available treatment for the most severe and impaired patients with OCD. Another significance of predictors of intensive treatment is that they might be used to improve intensive treatment by tailoring it to the characteristics of the patients who need it.

The goal of the present study was to identify predictors of starting with intensive treatment. In the rest of the text, we will refer to this as “predictors of intensive treatment,” for reasons of readability. We have selected potential predictors based on the above presented research findings in other populations. In addition, potential predictors were selected that have been associated with course and severity of OCD, leading to the following potential predictors: sociodemographic variables, clinical variables, and psychosocial variables including personality traits (25, 26), the quality of the social network (27), and childhood trauma (28–30). We hypothesized that being male, being younger, having more severe symptoms, poor insight in OCD, childhood trauma and a lower quality of social relationships are predictors of intensive treatment.

## Methods

The reporting of this study conforms to the STROBE statement (www.strobe-statement.org).

### Procedure

Data were derived from the Netherlands Obsessive Compulsive Disorder Association (NOCDA) study, longitudinal cohort study investigating the naturalistic long-term course of OCD in patients referred to mental health care centers. The NOCDA study design and baseline characteristics of the study sample are described in detail elsewhere (31). The NOCDA study was accredited by the Medical Ethical Committee of the VU-University Medical Center in 2005.

After their clinical assessment at one of the contributing mental health clinics, 687 patients aged 18 years and over with a lifetime diagnosis of OCD, as determined by the administration of the Structured Clinical Interview for DSM-IV Axis I Disorders (SCID-I) (32), were asked to participate in the NOCDA study. Since NOCDA aims to follow a large representative sample of OCD subjects in different stages of the disease and with different degrees of illness severity, the only exclusion criterion was an inadequate understanding of the Dutch language for the purposes of the completion of interviews and self-report questionnaires. Comprehensive measurements were done at baseline and after 2, 4, and 6 years.

Of the 687 patients who were asked to participate in the NOCDA study, 419 (60.9%) gave written informed consent and were enrolled in the study. A comparison on basic demographic characteristics between patients that did (*n* = 419) and did not (*n* = 268) agree to participate yielded no significant differences.

Baseline measurements took place between 2005 and 2009 and included validated semi-structured interviews and self-report questionnaires to gather information on a broad range of variables related to OCD, comorbidity, and psychosocial consequences. The baseline assessment took about 5 h. All included participants were contacted after 2, 4, and 6 years for assessment, irrespective of their treatment status. The follow-up assessments took about 3 h and in most cases (80%) they were performed by the same research assistant. During the follow-up period, participants received treatment as usual. Three hundred and eleven patients participated in the 2-year assessment (total dropout 26%), 295 patients in the 4-year assessment (total dropout 30%), and 272 patients in the 6-year assessment (total dropout 35%).

### Primary Outcome Measure: TIC-P

Treatment intensity was derived from the Treatment Inventory of Costs in Patients with mental disorders (TIC-P) (33). This is a 15-item interview assessing health care consumption in the previous 6 months (at baseline) or since the previous interview (at 2-, 4-, and 6-year). Treatment was scored as “intensive” when patients responded on the TIC-P interview (33) by stating that they were receiving day-care treatment or inpatient treatment in a psychiatric hospital or a specialized OCD clinic. In all other cases, treatment was scored as “not intensive.”

### Potential Predictors of Intensive Treatment in Patients With OCD

We studied three categories of potential predictors: sociodemographic, clinical, and psychosocial characteristics.

#### Sociodemographic Characteristics

Age (in years), gender, having a partner (yes, no), having children (yes, no), independent living situation [yes (living alone, with partner or children), no (living in a mental health institution or with parents)], education (number of years), paid employment (yes, no), and income (16 categories of increasing income).

#### Clinical Characteristics

Severity of OCD was assessed using the Yale Brown Obsessive Compulsive Scale for Severity (Y-BOCS) (34, 35). Age of onset of OCD was assessed using the SCID-I as the earliest age at which patients fulfilled the criteria for OCD. In order to assess the number of current comorbid mental disorders, the ascertained diagnoses on the SCID-I were counted (anxiety-, mood-, post-traumatic stress-, eating-, somatoform-, and substance-related disorders, and psychotic disorders). Presence and severity of comorbid anxiety symptoms were assessed using the Beck Anxiety Inventory (BAI) (36), while comorbid depressive symptoms were measured using the Beck Depression Inventory (BDI) (37–39). Psychotropic medication was assessed using the TIC-P (33), measuring use of all types of psychotropic medication in the previous 6 months (at baseline and follow-up). Insight in OCD was measured using the Overvalued Ideas Scale (OVIS) (40).

#### Psychosocial Characteristics

Childhood trauma was assessed using the Structured Trauma Interview (STI) (41). Traumas on the STI are: (1) early separation from parent; (2) and (3) parental dysfunction of mother or father respectively; (4) witnessing of interparental violence; (5) physical abuse; (6) sexual abuse. Ascertained childhood traumas were summed. Personality characteristics according to the Big Five were assessed using the Five-Factor Personality Inventory (FFPI) (42). Subscales of the FFPI are: extraversion, agreeableness, conscientiousness, emotional stability, and autonomy. Social support was assessed using the Social Support Inventory (43). The self-rated EuroQol five dimensional questionnaire (EQ-5D) was used to assess quality of life (44). The EQ-5D contains five dimensions significant for quality of life: mobility, selfcare, daily activities, pain/discomfort, and depression/anxiety.

Stable characteristics like age, gender, age of onset OCD, childhood trauma, and personality characteristics were assessed at baseline only. Characteristics that could vary over time were assessed at baseline, 2- and 4-year assessment. These characteristics were: relationship status, children, living situation, education, employment, severity of OCD, number of current comorbid mental disorders, comorbid anxiety and depressive symptoms, use of psychotropic medication, social support, and quality of life. An exception is the characteristic insight in OCD, which was assessed at 2- and 4-year assessment.

### Quality Aspects of NOCDA

The NOCDA study was coordinated by the Department of Psychiatry at the Amsterdam UMC/GGZ inGeest, Amsterdam, and included seven sites that were specialized OCD mental health clinics spread over the Netherlands. All research assistants had extensive experience in assessing OCD. In addition, they received a 2-day course, and regular follow-up 1-day training sessions in which videos of the SCID were rated, assessor rating scales were practiced, and questions and problems raised by the research assistants were able to be addressed. The first two interviews of all research assistants were audiotaped and monitored by the fieldwork coordinator in order to address any misunderstandings or errors in performing the measurements. All subsequent interviews were audiotaped for future reference. The audiotapes were continuously randomly monitored in about 10% of all taped interviews, as well as on the basis of questions raised by the research assistants and the fieldwork coordinator. Assessments were done by around 30 research assistants (profession: psychologist or experienced research nursing staff).

### Statistical Analyses

**Figure 1:**
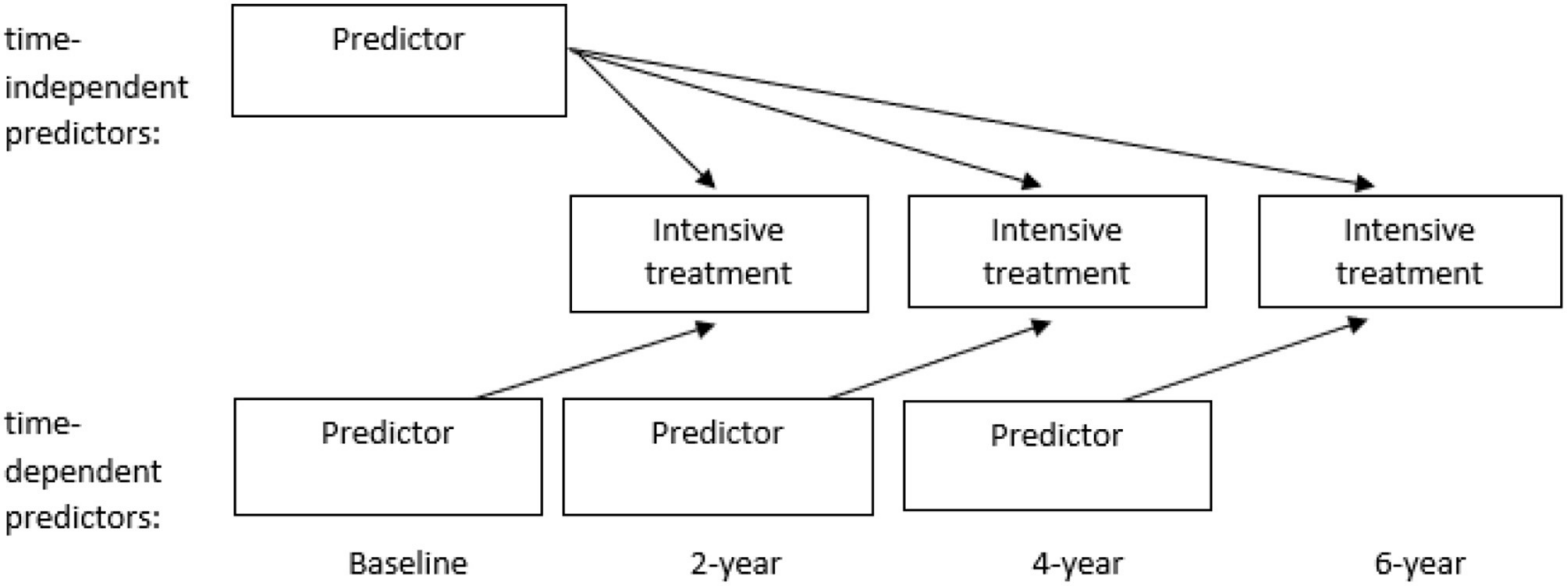
Schematic representation of statistical analyses.

Logistic Generalized Estimating Equations (GEE) with an exchangeable correlation structure was used to estimate to what extent the various characteristics (at baseline, 2- and 4-year assessment) predicted intensive treatment in the following period of 2 years, averaged over the three assessment periods (see Figure 1).

The following GEE analyses were performed: (1) univariable analyses in which all potential predictors were analyzed separately; (2) multivariable analyses within the three categories of potential predictors in which all variables of a category showing statistical significance in the univariable analyses were analyzed together; and (3) a multivariable analysis over the three categories including all variables showing statistical significance (*p* < 0.05) in the multivariable analyses within the three categories of potential predictors. A backward selection strategy was used to obtain the final multivariable models.

As a sensitivity analysis, we repeated the analyses but corrected for Y-BOCS severity. Insight in OCD will be analyzed separately using the other characteristics because it was assessed at 2- and 4-year assessment only.

## Results

### Description of Potential Predictors of Intensive Treatment

Table 1 presents the description of the potential predictors at baseline, 2- and 4-year measurement that may predict whether patients will receive intensive treatment in the following 2 years. The mean severity of OCD and comorbid symptoms decreased from baseline to 2-year measurement. From 2- to 4-year measurement, these severity scores stabilized or increased slightly.

**Table 1:** Descriptives of potential predictors of IT in patients with OCD.

**Potential predictor**	**Instrument range *min-max***	**Baseline *mean* (*SD*) or %**	**2-year measurement *mean (SD)* or *%***	**4-year measurement*mean (SD)* or *%***
		***n* = 419**	***n* = 311**	***n* = 295**
**Sociodemographics**				
Age, years	18–79^a^	36.6 (10.9)		
Gender, female		56%		
Partner, yes		62%	69%	66%
Child(ren), yes		37%	40%	55%
Living independently, yes		87%	95%	97%
Education, years	5–18^a^	12.6 (3.3)	13.2 (3.2)	13.2 (3.3)
Employment, yes		53%	60%	55%
Income	1–16	7.8 (4.2)	8.8 (4.6)	9.0 (4.8)
**Clinical characteristics**				
Y-BOCS obsessions	0–20	9.9 (4.3)	7.4 (4.8)	7.5 (4.7)
Y-BOCS compulsions	0–20	10.0 (4.8)	7.7 (5.0)	7.9 (5.2)
Y-BOCS total	0–40	19.9 (8.1)	15.1 (9.0)	15.4 (9.2)
Late age of onset OCD, yes^b^		39%		
Insight in OCD^c^	0–10	NA^d^	4.3 (1.5)	4.4 (1.3)
Comorbid disorders^e^	0–7^a^	1.8 (1.2)	1.2 (1.1)	1.2 (1.1)
Comorbid anxiety^f^	0–63	17.3 (12.0)	13.4 (11.2)	11.6 (9.8)
Comorbid depression^g^	0–40	15.3 (10.1)	11.6 (10.1)	13.6 (10.9)
Psychiatric medication, yes		75%	69%	70%
**Psychosocial characteristics**				
Extraversion^h^	−5.0–5.0	−0.07 (1.3)		
Agreeableness^h^	−5.0–5.0	2.2 (1.7)		
Conscientiousness^h^	−5.0–5.0	0.9 (1.5)		
Emotional stability^h^	−5.0–5.0	−0.7 (1.2)		
Autonomy^h^	−5.0–5.0	0.9 (1.1)		
Childhood trauma^i^	0–6	1.5 (1.2)		
Social support^j^	20–60	50.0 (8.4)	51.2 (9.3)	51.0 (9.2)
Quality of life^k^	0–1	0.6 (0.3)	0.8 (0.2)	0.7 (0.3)

a *Range in dataset*.

b *Onset ≥20 years*.

c *Overvalued Ideas Scale*.

d *Not available*.

e *Number of current comorbid psychiatric disorders*.

f *Beck Anxiety Index*.

g *Beck Depression Inventory*.

h *Subscale of Five-Factor Personality Inventory (FFPI)*.

i *Structured Trauma Interview*.

j *Social Support Inventory*.

k *EQ-5D utility score*.

### Description of 6-Year Course of Intensive Treatment

Table 2 presents the description of the intensive treatment variable over the course of 6 years. Over time, fewer patients were treated in mental health care (outpatient care as well as intensive treatment).

**Table 2:** Treatment of patients with OCD over the course of 6 years.

**Treatment**	**Baseline^**a**^**	**2-year^**b**^**	**4-year^**b**^**	**6-year^**b**^**
**Intensive treatment**				
Number of days				
*Mean (SD)*	55.8 (46.4)	96.3 (103.3)	168.9 (183.5)	78.7 (143.3)
*Median*	40	55	80	30
*n*	105 (25%)	76 (24%)	40 (14%)	30 (11%)
**Outpatient treatment**				
Number of sessions				
*Mean (SD)*	10.2 (8.7)	30.1 (29.0)	24.9 (26.6)	23.2 (22.0)
*Median*	7	21	16	18
*n*	287 (68%)	194 (62%)	166 (56%)	143 (53%)
**No treatment**				
*n*	26 (6%)	38 (12%)	88 (30%)	95 (35%)
**Missing**	1 (0%)	3 (1%)	1 (0%)	4 (1%)
*n*	419	311	295	272

a *Treatment in the previous 6 months*.

b *Treatment in the previous 2 years*.

### GEE Regression Analyses: Potential Predictors of Intensive Treatment

Table 3 presents the results of the analyses of the potential predictors of intensive treatment 2 years later over a time period of 6 years.

**Table 3:** Results of logistic GEE analyses of potential predictors of intensive treatment 2 years later over a time-period of 6 years.

	**Univariable analyses**			**Multivariable analyses within categories**			**Multivariable analyses over categories model 1**^**a**^			**Multivariable analyses over categories model 2**^**b**^		
	**OR**	**95% CI**	***p***	**OR**	**95% CI**	***p***	**OR**	**95% CI**	***p***	**OR**	**95% CI**	***p***
**Sociodemographics**												
Age, years	0.99	0.98, 1.01	0.30									
Gender, female	1.17	0.83, 1.65	0.38									
Partner, yes	0.54	0.44, 0.68	<0.01^*^	0.57	0.45, 0.71	<0.01^*^	0.62	0.51, 0.76	<0.01^*^	0.62	0.49, 0.77	<0.01^*^
Child(ren), yes	1.33	0.92, 1.92	0.12									
Living independently, yes	0.51	0.31, 0.84	0.01^*^									
Education, years	0.93	0.89, 0.98	0.01^*^									
Employment, yes	0.44	0.30, 0.64	<0.01^*^	0.50	0.45, 0.71	<0.01^*^						
Income	0.97	0.93, 1.01	0.12									
**Clinical characteristics**												
Y-BOCS total	1.10	1.06, 1.14	<0.01^*^									
Late age of onset OCD, yes^c^	1.01	0.84, 1.20	0.95									
Comorbid disorders^d^	1.30	1.19, 1.43	<0.01^*^									
Comorbid anxiety^e^	1.04	1.02, 1.05	<0.01^*^									
Comorbid depression^f^	1.05	1.04, 1.06	<0.01^*^	1.05	1.04, 1.06	<0.01^*^	1.04	1.03, 1.05	<0.01^*^			
Psychiatric medication, yes	2.75	1.62, 4.68	<0.01^*^	2.11	1.22, 3.63	0.01^*^	2.02	1.17, 3.48	0.01^*^	2.16	1.25, 3.74	0.01^*^
**Psychosocial characteristics**												
Extraversion^g^	0.81	0.70, 0.93	<0.01^*^									
Agreeableness^g^	0.96	0.80, 1.16	0.70									
Conscientiousness^g^	1.03	0.88, 1.21	0.74									
Emotional stability^g^	0.88	0.75, 1.02	0.10									
Autonomy^g^	0.84	0.72, 0.97	0.02^*^									
Childhood trauma^h^	1.14	0.99, 1.30	0.07									
Social support^i^	0.98	0.96, 0.99	0.01^*^									
Quality of life^j^	0.23	0.14, 0.38	<0.01^*^	0.23	0.14, 0.38	<0.01^*^				0.29	0.19, 0.46	<0.01^*^

a *Quality of life was omitted in model 1 due to multicollinearity between comorbid depression and quality of life*.

b *Comorbid depression was omitted in model 2 due to multicollinearity between comorbid depression and quality of life*.

c *Onset ≥20 years*.

d *Number of current comorbid psychiatric disorders*.

e *Beck Anxiety Index*.

f *Beck Depression Inventory*.

g *Subscale of Five-Factor Personality Inventory (FFPI)*.

h *Structured Trauma Interview*.

i *Social Support Inventory*.

j *EQ-5D utility score*.

* *p < 0.05*.

In the univariable analyses, not having a partner, a dependent living situation, fewer years of education, not having a paid job, more severe OCD, more current comorbid diagnoses, more severe comorbid anxiety and depression, use of psychotropic medication, less extraversion, less autonomy, less social support, and a lower quality of life all significantly predicted intensive treatment 2 years later.

In the multivariable analysis of the sociodemographic variables, not having a partner and not having a paid job significantly predicted intensive treatment 2 years later. Predictors in the multivariable analysis of the clinical variables were more severe comorbid depression and use of psychotropic medication, while in the multivariable analysis of the psychosocial variables a lower quality of life predicted intensive treatment 2 years later.

For the final multivariable model, in which all significant predictors from the previous multivariable models were analyzed together, severity of comorbid depression and quality of life could not be included together due to high collinearity. Because severity of comorbid depression had a stronger association with intensive treatment, a final multivariable model was made with this variable and quality of life was not included (Table 3 model 1). In this model, not having a partner, more severe comorbid depression and use of psychotropic medication significantly predicted intensive treatment 2 years later. When quality of life was substituted for severity of comorbid depression in the final multivariable model (Table 3 model 2), it appeared that a lower quality of life significantly predicted intensive treatment 2 years later as well as not having a partner and use of psychotropic medication.

From the sensitivity analysis, in which we repeated the analyses but corrected for Y-BOCS severity, it appeared that the same predictors were significantly related to intensive treatment in the final multivariable analysis. Thus, these factors predict intensive treatment independently of OCD severity.

Insight in OCD was not significantly related to intensive treatment [OR = 1.07, 95% CI (0.98, 1.18); *p* = 0.14].

## Discussion

We studied potential predictors of intensive treatment in the subsequent 2 years in patients with OCD over the course of 6 years. It appeared that patients with OCD who were single, who had more severe comorbid depressive symptoms, who used psychotropic medication, and who had a low quality of life were significantly more likely to have intensive treatment 2 years later. Our results on being single and more severe comorbid depression resemble the results concerning other mental disorders (18, 19, 21). Thus, also in patients with OCD, these variables predict future intensive treatment. Quality of life as a potential predictor of intensive treatment has not been studied before. Our result that psychotropic medication predicts future intensive treatment is not congruent with previous research results in which a negative attitude toward medication—and thus likely not using medication—predicted admission (18, 22). This difference might reflect the different study populations. While in patients with psychotic disorders or mood disorders medication has a large effect on symptoms and prevents relapse, crisis, and hospitalization (45, 46), in OCD, medication has only a moderate effect. SSRIs cause a mean reduction of 3.2 points on the Y-BOCS, over placebo, in patients with OCD according to a meta-analysis including 17 studies (3,097 participants) (47). Therefore, patients with OCD not taking medication usually does not lead to severe relapse or crisis, or an increase in the risk of hospitalization. A second explanation for our finding might be that stepped-care principles were followed in the treatment of OCD that indicate prescription of psychotropic medication before stepping up to more intensive treatments (3).

Contradictory to our hypotheses, the following potential predictors did not significantly predict intensive treatment. Remarkably, although severity of OCD was associated with intensive treatment in the univariable analysis of our study, this association disappeared in the multivariable models, indicating that other variables were more important in predicting intensive treatment. This might indicate that despair and limitations as a result of OCD are more important reasons for intensive treatment than severity of OCD *per se*. Next, insight in OCD did not predict intensive treatment in our study. This is not congruent with a previous finding in patients with several mental disorders that better insight was predictive of readmission (48). Also, it is not in line with previous findings that patients with poor insight in OCD were less likely to seek mental health care (49). In addition, poor insight in OCD was previously related to severity and chronicity of OCD (50–52). Possibly, effects of insight and help-seeking on intensive treatment cancel each other out. More specifically, patients with poor insight are often severe and chronic patients for whom intensive treatment is indicated. However, they are less likely to seek help. Conversely, patients with good insight do seek help but need intensive treatment less often. Lastly, childhood trauma was not predictive of intensive treatment in our study. To our knowledge, childhood trauma has not been studied as a potential predictor for intensive treatment before. Contradictory results have been found on the association between childhood trauma and severity and chronicity of OCD (28–30, 53). While childhood trauma is an important predictor of severity and chronicity of depression in patients with depressive disorders, the relationship between childhood trauma and severity and chronicity of OCD is less clear (54).

The predictors of intensive treatment that have emerged from our study might be used to tailor intensive treatment to the characteristics of the patients involved. For instance, single patients obviously lack the support from a partner, which might make it harder for them to stay motivated in the face of setbacks in treatment. Therapists may need to organize or offer extra support to pull these patients through. Next, patients with comorbid depressive symptoms or with a low quality of life may have difficulty following an intensive treatment program. In that case, adapting the treatment to the impairment of the patient may be helpful, and could be done by including activation in treatment or by shortening treatment days.

Our results indicate that intensive treatment might be prevented by improving comorbid depression and quality of life in first-step treatments in addition to treating OCD. In other words, to not focus only on diminishing OCD symptoms in treatment but also on vitality and promoting a fulfilling life with elements that patients want from life, like work, pleasurable activities, a partner, and a social network. We recommend therapists to encourage patients to fulfill life's wishes while allowing them to be hindered by OCD as little as possible. In our clinical experience, patients tend to postpone fulfilling their life's wishes based on the idea that it is better to wait until the OCD symptoms have disappeared. However, this conviction contributes to the notion of being disabled, which drives patients further away from their goals in life and in treatment. Therefore, therapists should educate patients about the importance of working on their life's goals in treatment in addition to working on OCD. Furthermore, therapists can help to find practical solutions to obstacles that may arise.

During the last decade, it has been accepted that recovery from mental disorders does not just entail having fewer symptoms but also regaining functioning and resuming a meaningful life (55). Guidelines like the NICE and the APA guidelines recognize the importance of focusing on functioning and quality of life in treatment (3, 4). Also, treatments are increasingly being evaluated using quality of life outcome measures (56–58). Moreover, recovery-oriented treatment programs have been implemented for patients with severe mental illness like schizophrenia, bipolar disorder, major depressive disorder, borderline personality disorder, and substance use disorders. These treatment programs foster adapting to chronic mental illness and movement toward personally meaningful goals like work and education (59–61). Recovery-oriented treatment programs help to improve both symptoms and functioning and help reduce hospitalization in these patients with severe mental illness (59). Another treatment that can be effective in improving quality of life is acceptance and commitment therapy (ACT), which aims to accept negative feelings, while moving toward meaningful goals in accordance with personal values (62–64).

A limitation of this study is that although we had a longitudinal study design with potential predictors preceding the outcome measure (intensive treatment) in time, we were unable to establish causal connections between potential predictors and intensive treatment. Future research should thus examine whether treatment of the significantly associated predictors of our study indeed prevents intensive treatment. Another limitation is the attrition rate of 35% over the course of 6 years. To investigate whether dropout was selective, we have compared baseline characteristics of patients who participated in the 6-year assessment with patients who did not participate. Patients did not differ on any of the baseline characteristics except that patients who dropped out had less years of education (mean = 11.7; SD = 3.3) compared to patients who participated in the 6-year assessment [mean = 13.1; SD = 3.2; *t*_(416)_ = −4.2, *p* < 0.01]. In previous studies, education was a determinant of attrition as well (65, 66). Presumably, our results were not biased by selective attrition. Last limitation is a potential historical effect due to the fact that the data was collected between 2005 and 2015. However, the intensive treatments that were common in the Netherlands during the NOCDA data collection have largely remained the same to date. This study also had a strength: we had access to a large, representative sample of treatment-seeking patients with OCD who were followed for a long period of time. Thus, our results are generalizable to clinically referred OCD patients in a specialized setting.

In conclusion, therapists should be aware that patients with OCD who are single, who have more severe comorbid depression, who use psychotropic medication, and who have a low quality of life or a drop in quality of life are at risk for intensive treatment. This is significant because knowledge of these predictors might help to optimize first-step treatments for patients with OCD to prevent the necessity of intensive treatment. In addition, the significant predictors of our study might be used to tailor intensive treatment to the characteristics of patients involved. We advise working on comorbid depression and personal goals in treatment in addition to working on OCD. Also, we advise providing extra support in treatment for patients who need it and to adjust treatment to impairments due to comorbid depressive symptoms or a low quality of life.

## Data Availability Statement

The datasets presented in this article are not readily available because of privacy restrictions. Requests to access the datasets should be directed to the authors and the datasets will be made available after a data analysis plan is approved and a data sharing form is filled out.

## Ethics Statement

The studies involving human participants were reviewed and approved by Medical Ethical Committee of the VU-University Medical Centre. The patients/participants provided their written informed consent to participate in this study.

## Author Contributions

JdM and KR designed the study, wrote the manuscript and performed the literature search. NB, AvB, and HV designed the study and supervised all aspects of this study. PvO organized the database and had significant input on the text. JT designed the study and performed the statistical analysis. All authors contributed to manuscript revision, read, and approved the submitted version.

## Conflict of Interest

The authors declare that the research was conducted in the absence of any commercial or financial relationships that could be construed as a potential conflict of interest.
